# A novel method for estimating pathogen presence, prevalence, load, and dynamics at multiple scales

**DOI:** 10.1038/s41598-025-93865-x

**Published:** 2025-03-19

**Authors:** John F. Grider, Bradley J Udell, Brian E. Reichert, Jeffrey T. Foster, William L. Kendall, Tina L. Cheng, Winifred F. Frick

**Affiliations:** 1 Colorado Cooperative Fish and Wildlife Research Unit, Colorado State University, 1484 Campus Delivery, Fort Collins, CO 80523 USA; 2 Colorado Parks and Wildlife, Wildlife Health Program, 4330 Laporte Avenue, Fort Collins, CO 80521 USA; 3 Fort Collins Science Center, U.S. Geological Survey, 2150 Centre Avenue, Fort Collins, CO 80526 USA; 4 Pathogen and Microbiome Institute, Northern Arizona University, Flagstaff, AZ 86011 USA; 5 Colorado Cooperative Fish and Wildlife Research Unit, U. S. Geological Survey, Colorado State University, 1484 Campus Delivery, Fort Collins, CO 80523 USA; 6 Bat Conservation International, 500 North Capital of Texas Highway, Building 1, Austin, TX 78746 USA; 7 Department of Ecology and Evolution, University of California, 130 McAllister Way, Santa Cruz, Santa Cruz, CA 95060 USA

**Keywords:** Cycle threshold (Ct), Little brown Bat, Multi-scale modeling, Pathogen load, Quantitative polymerase chain reaction (qPCR), White-nose syndrome, Ecology, Diseases

## Abstract

The use of quantitative real-time PCR (qPCR) to monitor pathogens is common; however, quantitative frameworks that consider the observation process, dynamics in pathogen presence, and pathogen load are lacking. This can be problematic in the early stages of disease progression, where low level detections may be treated as ‘inconclusive’ and excluded from analyses. Alternatively, a framework that accounts for imperfect detection would provide more robust inferences. To better estimate pathogen dynamics, we developed a hierarchical multi-scale dynamic occupancy hurdle model (MS-DOHM). The model used data gathered during sampling for *Pseudogymnoascus destructans (Pd)*, the causative agent of white-nose syndrome, a fungal disease that has cause severe declines in several species of hibernating bats in North America. The model allowed us to estimate initial occupancy, colonization, persistence and prevalence of *Pd* at bat hibernacula. Additionally, utilizing the relationship between cycle threshold and pathogen load, we estimated pathogen detectability and modeled expected colony and bat pathogen loads. To assess the ability of MS-DOHM to estimate pathogen dynamics, we compared MS-DOHM’s results to those of a dynamic occupancy model and naïve detection/non-detection. MS-DOHM’s estimates of site-level pathogen presence were up to 11.9% higher than estimates from the dynamic occupancy model and 35.7% higher than naïve occupancy. Including prevalence and load in our modeling framework resulted in estimates of pathogen arrival that were two to three years earlier compared to the dynamic occupancy and naïve detection/non-detection, respectively. Compared to naïve values, MS-DOHM predicted greater pathogen loads on colonies; however, we found no difference between model estimates and naïve values of prevalence. While the model predicted no declines in site-level prevalence, there were instances where pathogen load decreased in colonies that had been *Pd* positive for longer periods of time. Our findings demonstrate that accounting for pathogen load and prevalence at multiple scales changes our understanding of *Pd* dynamics, potentially allowing earlier conservation intervention. Additionally, we found that accounting for pathogen load and prevalence within hibernacula and among individuals resulted in a better fitting model with greater predictive ability.

**Supplementary Information:**

The online version contains supplementary material available at 10.1038/s41598-025-93865-x.

## Introduction

Responding rapidly to emerging disease depends on reliable surveillance methods capable of detecting pathogens as they spread^1,2^. Monitoring methods using DNA are an increasingly common tool for sampling organisms due to their ability to detect cryptic species at low densities, their non-invasive nature, and the relatively cost-effective, easy, and reproducible ways they can be deployed^3^. While generally applied in a detection/non-detection estimation framework, when paired with quantitative real-time PCR (qPCR), we can use cycle thresholds (Ct values) as an estimate of DNA load (relative amount of DNA in a sample) to inform detection and as a measure of pathogen quantity^4,5^. The use of qPCR allows new methods for analyzing outputs that account for detection issues, spatial and temporal variation, population dynamics, and the relationship between DNA load and pathogen abundance^6–9^. While existing methods have proven useful, integrating information from qPCR sampling into a hierarchical and holistic modeling framework can improve species monitoring efforts, including pathogens, and inform conservation responses in real time^3^.

In North America, the introduction of *Pseudogymnoascus destructans *(*Pd*), the fungal pathogen that causes the disease white-nose syndrome (WNS) in bats, has caused mass mortality leading to greater than 90% population declines in several bat species^10^. The *Pd* fungus invades the skin tissue of bats^11–13^ during winter when bats are hibernating^14^. The fungal infection disrupts normal hibernation behavior and physiology resulting in high mortality during winter^15,16^. Since the disease was first observed and the causative pathogen identified^17^, there has been considerable research conducted to describe the pathogen, its associated disease, and develop an effective surveillance plan^18–20^. The aim of *Pd* surveillance efforts has primarily been to monitor and report pathogen spread to inform disease response efforts^21^.

The standard approach to sample for *Pd* is conducted by swab-sampling bats and their roosting substrates during hibernation^14,18,19,22^, which is then analyzed using qPCR to detect pathogen presence and estimate pathogen loads^23^. Presence of the pathogen is determined based on cycle thresholds (Ct) from qPCR, which represents the number of cycle amplifications needed to detect the target nucleic acid in a sample and is often used as a relatively quick way to assess qPCR results^24^. Currently, *Pd* surveillance guidance from the White-nose Syndrome Response Team (www.whitenosesyndrome.org) recommends use of Ct scores to classify *Pd* presence as positive, negative, or inconclusive^25^. Amplification of DNA at high Ct scores typically indicates low-level positives but because these results can also arise from amplification of non-target DNA or contamination^26^, these results are often deemed “inconclusive” rather than positive. Inconclusive designations from qPCR diagnostic testing are used in other disease systems, including the recent SARS-COV2 pandemic^27^. While “inconclusive” designations can be useful in characterizing uncertainty when interpreting results, the use of this term can create confusion among clinicians regarding how to interpret an “inconclusive” result and could lead to delayed management^28^. Conversely, treating all inconclusive tests as positives could lead to wasted resources and misconceptions surrounding the pathogen dynamics. A potential solution circumventing an “inconclusive” designation is to build detection uncertainty from qPCR outputs into a modeling framework that could better distinguish low-level detections and facilitate early warning of *Pd* invasion to new sites.

Sources of uncertainty in pathogen detection can occur during the selection of spatial units and individuals for sampling, false positives and negatives in laboratory assays, and biotic and abiotic factors^18,29–31^ When sampling for *Pd*, detection can vary temporally, with mid and late winter hibernacula surveys being optimal due to *Pd* prevalence and fungal loads increasing throughout winter^14,18,19^. Pathogen detection can also vary by type of sample collected, with detection on bats preceding detection on substrate, and *Pd* often more readily detected in sediment^32^. In the laboratory, pathogen detection can potentially vary from deficiencies in qPCR methods and techniques, resulting in false positives or negatives^33–35^. Despite these possible sources of error and variation in *Pd* sampling, most analyses do not account for potential misclassification of pathogen presence, which can lead to uncertainty surrounding disease presence and progression^36,37^.

The continental surveillance effort for *Pd* has largely focused on and reported instances of disease occurrence or pathogen presence^21^. Using qPCR outputs can provide more information than just *Pd* presence and utilizing pathogen prevalence and loads from sampled colonies could prove informative for management. However, reports of these metrics are often limited to researcher’s efforts, who choose to contribute information to the response effort, but are not explicitly part of the continental surveillance plan^19,20^. Specifically, pathogen prevalence and loads on hosts and the environment can inform how the disease is progressing through a local population, how mortality from the disease may vary spatially and temporally, and how the host responds to infection^38,39^. For bats in North America, *Pd* prevalence and load typically increase for several years following initial detection, with highly susceptible species experiencing a rapid increase in both prevalence and load both during winter for several years^19,32^.

We examined how integrating multiple levels of data from a continental surveillance effort for the *Pd* pathogen can improve our understanding of the spread and progression of WNS disease in hibernating bats. Specifically, we developed a novel multi-scale dynamic occupancy hurdle model (MS-DOHMS) that accounts for detection probability and estimates pathogen presence, prevalence, and load annually at hibernacula^40,41^. Currently, the continental surveillance effort uses naïve detection/non-detection to determine *Pd* presence and does not utilize model-based inferences^21^. Furthermore, current occupancy methodology does not fully utilize continuous data like load to inform detection probabilities or define a continuous infection intensity state. We compared model outputs and estimates from the MS-DOHMS approach to that of a dynamic occupancy model and naïve detection/non-detection to determine how this approach could be used to improve our understanding of *Pd* at scales relevant for management response.

## Methods

### Field sampling

We used data collected as part of a large collaborative study conducted from 2011 to 2017 that involved coordinated sampling across continental North America to understand the pathogen dynamics and effect of WNS on bats^19^. For the purposes of this analysis, we used a subset of the data to include sites with hibernating little brown bats (*Myotis lucifugus*). We used samples collected in 42 caves, mines, tunnels, and culverts used as hibernacula. Each site was sampled for a minimum of two winters and an average of 3.5. During each visit, we sampled *M. lucifugus* by dipping a sterile polyester swab in sterile water and making five passes over the epidermis of the bat’s forearm and muzzle^14^. We collected an average of 11 swabs (range: 1–61) from *M. lucifugus* during each site visit. We stored swabs in RNAlater until extraction and tested for presence and quantity of *Pseudogymnoascus destructans* DNA using qPCR^23^. We conducted all animal testing in accordance with the relevant regulations including IACUC approval (Frickw1405) issued by UC Santa Cruz and ARRIVE guidelines. We released all sampled individuals alive on site.

### qPCR analysis

Using qPCR, we ran each swab in either duplicate or triplicate, with an average of 2.1 runs per swab. We determined a run to be *Pd* positive based on Ct values ≤ 40. For data used in statistical models, we considered the sample *Pd* positive if at least one run resulted in a Ct ≤ 40. For naïve occupancy, we considered a Ct ≤ 37 in one run *Pd* positive. Naïve estimates of prevalence and load were derived by taking the proportion of swabs that tested positive (Ct ≤ 37) and the mean load across all positive swabs, respectively. We used a cutoff of 37 for naïve estimates as this is what is commonly used to delineate samples as positive (Ct ≤ 37), suspect (40 ≥ Ct > 37), and negative. We accounted for pathogen load by equating the Ct from a qPCR run to pathogen load. We related Ct to the starting amount of DNA in a run using a quantification curve from serial dilutions (nanograms of *Pd* = $\:{10}^{\left(\frac{\left(\text{C}\text{t}-\:22.04942\right)}{-3.34789}\:\right)}$;^18^). All qPCR tests were conducted in a single laboratory.

### Model structure

**Fig. 1:**
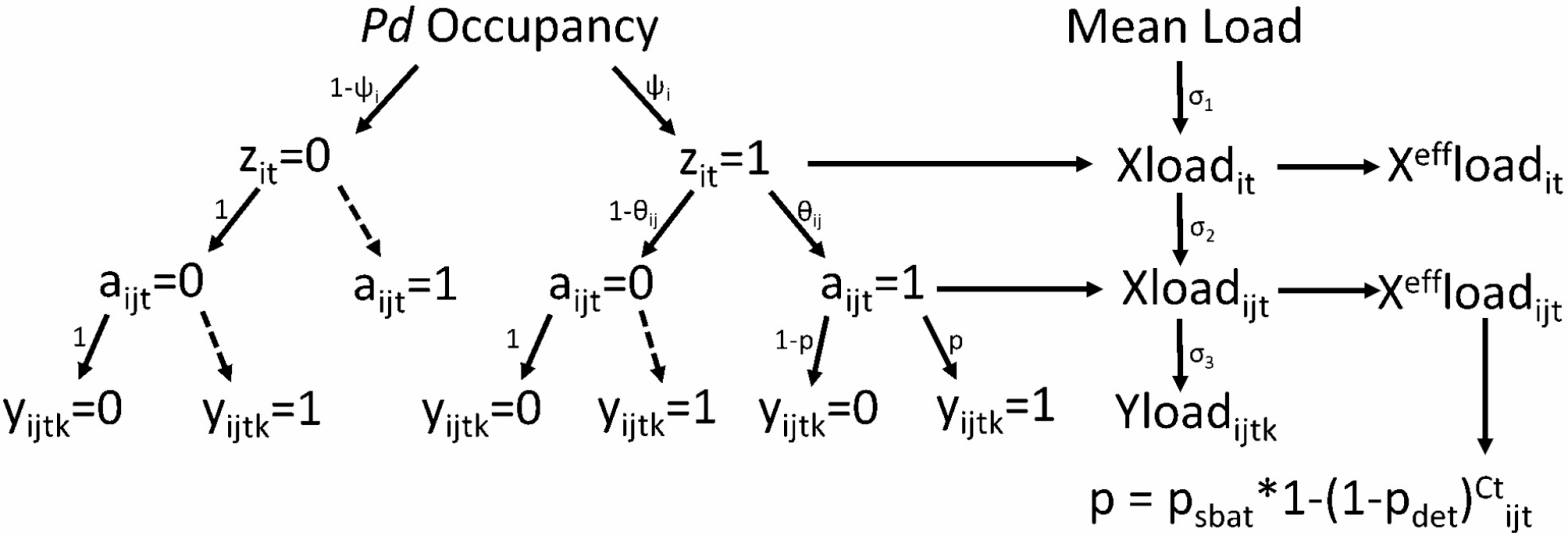
Conceptual diagram of how occupancy, prevalence, and load are estimates at the hibernaculum (i), swab (j), and run (k) level annually (t) using the multi-scale dynamic occupancy hurdle model. The model utilizes quantitative real-time PCR (qPCR) runs (y_ijtk_) from swabs (a_ijt_) used to determine the pathogen status of *Myotis lucifugus* at winter hibernacula (z_it_). Probability of detection (p) was modeled via the probability of detecting *Pseudogymnoascus destructans*(*Pd)* on a bat (p_sbat_) multiplied by the probability of *Pd* DNA replicating in the sample given it is present. Probability of the DNA replicating was dependent on the Cycle threshold (Ct) score and per unit (of *Pd* load) detection probability (p_det_) which is scaled by the latent average *Pd* load of each bat (x^eff^load_ijt_). We derived the load for each run (yload_ijtk_) by transforming the Ct score. We then calculated the average load per swab (xload_ijt_), hibernaculum (xload_it_), and across all hibernacula (Mean Load) by accounting for observation error due to load variation (σ_3_), load variation between individuals within a cave (σ_2_), and variation in load across caves (σ_1_), respectively. Effective loads (x^eff^load_it_ and x^eff^load_ijt_) are calculated by multiplying the hibernacula or swab status by load. Dashed lines indicate false positive outcomes, which we assumed did not occur.

We developed a hierarchical model and used Bayesian statistics to estimate pathogen occupancy (presence) among hibernacula of *M. lucifugus*, prevalence (proportion of *M. lucifugus* with *Pd* in occupied hibernaculum), and relative pathogen load on swabs used to sample the pathogen, individual *M. lucifugus*, and winter colonies of *M. lucifugus* at a given hibernacula (Fig. 1). The kernel of this model is a multiscale occupancy model^42,43^, where at time *t* each hibernaculum *i* is occupied by *Pd* with probability $\:{\psi\:}_{it}$, and for each occupied hibernaculum each bat therein is infected with prevalence probability $\:{\theta\:}_{it}.$ qPCR runs from each swab test positive for *Pd* with probability *p.* This multiscale structure is nested with a hurdle model that uses measures of pathogen load to inform presence of the pathogen in a qPCR run, and pathogen load at the individual bat and hibernaculum level (Fig. 1).

We let $\:{y}_{i,j,t,k}$ indicate the binary detection of *Pd* in a qPCR run (positive if Ct ≤ 40) and $\:{y}_{loa{d}_{i,j,t,k}}\:$indicate the measured *Pd* load on a swab run in a given colony (*i*), bat (*j*), year (*t*) and run (*k*). Further, we denote $\:{\text{p}}_{i,j,t}$ as the binary detection probability of *Pd* in a sample$\:\:{y}_{i,j,t,k}$, which varies by both a colony within hibernaculum (*i*), individual bat (*j*) and year (*t*). We hierarchically model the occupancy status of each *M. lucifugus* winter colony at the hibernaculum $\:{z}_{i,t}$ and bat $\:{a}_{i,j,t}\:$level within each hibernaculum given the occupancy probability of winter colony at a hibernaculum $\:{\psi\:}_{i,t}$ and the prevalence $\:{\theta\:}_{i,t}$ in each hibernaculum. For example, in the first year of the study, the occupancy state at each hibernaculum $\:{z}_{i,t=1}$is modeled based on a Bernoulli distribution given the occupancy probability in year 1 $\:{\psi\:}_{i,t=1}:$

$$\:{z}_{i,t=1}\sim\:\text{B}\text{e}\text{r}\text{n}\left({\psi\:}_{i,t=1}\right)$$

Then, occupancy at the bat level $\:{a}_{i,j,t=1}$in year 1 is modeled given the availability parameter (i.e., the prevalence given presence in the hibernacula $\:\left({\theta\:}_{i,t=1}\right))$ as:

$$\:{{\theta\:}^{*}}_{i,t=1}=\:{z}_{i,1}{\theta\:}_{i,t=1}$$

$$\:{a}_{i,j,t=1}|{z}_{i,t=1}\:\sim\:\text{B}\text{e}\text{r}\text{n}\left({{\theta\:}^{*}}_{i,t=1}\right)$$

And in all subsequent years as

$$\:{{\theta\:}^{*}}_{i,t}=\:{z}_{i,t}{\theta\:}_{i,t}$$

$$\:{a}_{i,j,t}|{z}_{i,t}\:\sim\:\text{B}\text{e}\text{r}\text{n}\left({{\theta\:}^{*}}_{i,t}\right)$$

We allowed occupancy and prevalence to update annually$\:\:\left(t\right)$, based on estimates of persistence$\:{\:\varPhi\:}_{it}$ and colonization $\:{{\rm\:Y}}_{it}\:\text{a}\text{t}\:\text{h}\text{i}\text{b}\text{e}\text{r}\text{n}\text{a}\text{c}\text{u}\text{l}\text{a}\:\left(h\right)\:\text{a}\text{n}\text{d}\:\text{i}\text{n}\text{d}\text{i}\text{v}\text{i}\text{d}\text{u}\text{a}\text{l}\:\text{b}\text{a}\text{t}\:\left(b\right)\:\text{l}\text{e}\text{v}\text{e}\text{l}$.

$$\:{z}_{i,t}\sim\:\text{B}\text{e}\text{r}\text{n}\left({z}_{i,t-1}{{\varPhi\:}^{h}}_{i,t}+(1-{z}_{i,t-1}){{{\rm\:Y}}^{h}}_{i,t}\right)$$

$$\:{{\theta\:}^{*}}_{i,t}=\:{z}_{i,t}\left({\theta\:}_{i,t-1}{{\varPhi\:}^{b}}_{i,t}\right)+(1-{\theta\:}_{i,t-1}){{{\rm\:Y}}^{b}}_{i,t}$$

The parameters $\:{\psi\:}_{i,t}$, $\:{\theta\:}_{i,t}$, $\:{\varPhi\:}_{i,t}$, and $\:{{\rm\:Y}}_{i,t}\:$can each be modeled as a function of covariates or random effects using a logit link function, if separate covariates can be identified to inform each. We included site-level random effects to all equations related to $\:{\psi\:}_{i,t}$ and $\:{\theta\:}_{i,t}$.

Finally, the binary detection process for *Pd* given occupancy state of each individual bat is the occupancy state of the bat times the probability of detection ($\:{\text{p}}_{i,j,t}$)

$$\:{y}_{i,j,t,k}\sim\:\text{B}\text{e}\text{r}\text{n}\left({a}_{i,j,t}\text{*}{\text{p}}_{i,j,t}\right)$$

We also modeled the measured *Pd* load ($\:{yload}_{i,j,t,k})$ at the colony (*i*), bat (*j*), year (*t*) and run (*k*) level hierarchically given the occupancy status of colonies and bats, and the *Pd* load given presence of each hibernaculum $\:\left(x{load}_{i,t}^{cave}\right)\:$and bat$\:\left(x{load}_{i,j,t}^{bat}\right)$ as a lognormal distribution (Fig. 1). For example, in year 1 measured *Pd* load given presence on the sampled bat and the latent *Pd* load $\:xloa{d}_{ba{t}_{i,j,t}}$ is

$$\:{yload}_{i,j,t=1,k}|x{load}_{i,j,t=1}^{bat}\sim\:\text{L}\text{o}\text{g}\text{n}\text{o}\text{r}\text{m}\text{a}\text{l}\left(\text{m}\text{e}\text{a}\text{n}=x{load}_{i,j,t=1}^{bat},\:\text{s}\text{d}={\sigma\:}_{\text{r}\text{u}\text{n}}\right)$$

While the latent *Pd* load of each of each bat, at each hibernacula each year $\:xloa{d}_{ba{t}_{i,j,t=1}}$ was modeled as

$$\:x{load}_{i,j,t=1}^{bat}|x{load}_{i,t}^{cave}\sim\:\text{L}\text{o}\text{g}\text{n}\text{o}\text{r}\text{m}\text{a}\text{l}\left(\text{m}\text{e}\text{a}\text{n}=x{load}_{i,t=1}^{cave},\:\text{s}\text{d}={\sigma\:}_{\text{b}\text{a}\text{t}}\right)$$

We modeled dynamics on loads for winter colonies at hibernacula $\:X{load}_{i,t}^{cave}$ by modeling the expected value at year 1 $\:\:{\mu\:}_{i,1}$ as a function of covariates, and then including a linear drift term $\:\varDelta\:{\mu\:}_{i}$ which was added to the prior year’s value of $\:X{load}_{i,t}^{cave}\:$on a log scale for every time step *t* as:.

$$\:\text{log}\left(x{load}_{i,t}^{cave}\right)\sim\:\:\text{N}\text{o}\text{r}\text{m}\text{a}\text{l}\left(\text{m}\text{e}\text{a}\text{n}=\:{\mu\:}_{i,t}\:,\:\text{s}\text{d}={\sigma\:}_{\text{h}\text{i}\text{b}}\right)$$ $$\:\varDelta\:{\mu\:}_{i}\:\sim\:\text{N}\text{o}\text{r}\text{m}\text{a}\text{l}\left(\text{m}\text{e}\text{a}\text{n}={r}_{\text{o}\text{v}\text{e}\text{r}\text{a}\text{l}\text{l}}\:,\:\text{s}\text{d}={\sigma\:}_{\text{h}\text{i}\text{b}2}\right)\:$$ $$\:\:{\mu\:}_{i,t+1}=\:\:{\mu\:}_{i,t}+\varDelta\:{\mu\:}_{i}\:$$

And the effective latent *Pd* load for each bat is$\:{x}^{eff}{load}_{i,j,t}^{bat}={a}_{i,j,t}\text{*}x{load}_{i,j,t}^{bat}$

And for each cave is

$$\:{x}^{eff}{load}_{i,t}^{cave}={\theta\:}_{i,t}\text{*}x{load}_{i,t}^{cave}$$

Together, these processes are a Bernoulli-lognormal hurdle model for *Pd* occupancy and *Pd* load given occupancy. Detectability of *Pd* in a sample and the *Pd* load of a sample should be related, with lower detectability at lower *Pd* loads (higher Ct). We model the detection probability of *Pd* in a sample based on the following relationship:

$$\:{\text{p}}_{i,j,t}={p}_{bat}*\left(1-{\left(1-{p}_{det}\right)}^{{(41-Ct\:score}_{{bat}_{i,j,t}})}\right)$$

Where $\:{p}_{bat}$ is the probability of detecting *Pd* on a bat and making it into the extraction, and $\:\left(1-{\left(1-{p}_{det}\right)}^{{(41-Ct\:score}_{{bat}_{i,j,t}})}\right)$ is the probability of the DNA replicating in the sample given it is present, where $\:{p}_{det}\:$is the per unit (of *Pd* load) detection probability which is scaled by the latent average *Pd* load of each bat. We assume there are no-false positives in this model, rather, all uncertainty deals with uncertain detection (i.e., detectability). We believe this assumption is justified for this data set, given the extensive use of negative controls (16 negative control wells per 96-well plate) used in the laboratory procedures and the lack of false positives in these controls in over 90,000 samples run.

### Covariates

We investigated the influence of space and time on pathogen occurrence, as previous research has correlated *Pd* spread spatially and temporally^19,44,45^. We defined each winter period as occurring from November 1st to April 15th, during which we assumed that sites were closed to *Pd* colonization or extinction^14^. We determined distance from the nearest *Pd* positive location for each time step by measuring the straight-line distance from each site to the nearest known *Pd* detection in the previous time step. We supplemented site specific locations from our data with centroids from counties/territories with positive *Pd* detection, based on the WNS occurrence map, at www.whitenosesyndrome.org. We applied model estimates of mean colony *Pd* load from the previous winter period to estimate the likelihood of the pathogen persisting at a site to the next winter. To account for potential differences in *Pd* presence and prevalence among hibernacula, we investigated the effect of year since initial *Pd* detection. We investigated if the presence of other susceptible species, particularly Myotis septentrionalis, *Perimyotis subflavus*, and *Myotis sodalis*, influenced site level prevalence^46^. We considered days since onset of hibernation and year since initial pathogen detection (Appendix A) as effects on bat *Pd* load and initial prevalence within hibernacula, since pathogen prevalence and load increase within winter seasons^19^.

### Model comparison

We ran all models in rjags using program R and vague priors^47,48^. To assess MS-DOHM’s ability to predict pathogen spread, we compared its results with those from naïve detection/non-detection data and a dynamic occupancy model^49^. Naïve occupancy and the dynamic occupancy model do not utilize count data, so we could only compare model inferences for site-level pathogen presence and prevalence. To test model fit, we conducted a posterior predictive check and calculated the Bayesian p-value by comparing chi squared fit statistics of the observed and simulated data, where a value of 0.5 indicates a perfect fit and values < 0.1 and > 0.9 suggest poor fit^50^. We constructed posterior summaries based on 15,000 Monte Carlo Markov Chain (MCMC) samples from 3 chains, thinned by a factor of 8, after and burn in of 20,000 and 20,000 adaptations. We assessed convergence of chains via trace plots and R-hat values, with convergence reached when |R-hat − 1| < 0.1. Unless otherwise noted, we report estimates of the posterior mean and 95% credible interval. To assess differences in prevalence and load between the MS-DOHM and naïve estimates we used paired t-tests.

### Simulations

To determine if the model could consistently and accurately estimate predictor variables, we simulated data and refit the simulated data to the MS-DOHM. The model used in data simulation contained a parameter on site-level colonization and extinction with all other portions of the model being intercept only (Appendix B). We used biologically appropriate parameter values to ensure the model returned simulated values in the range of our parameter estimates. For consistency with the case study, we simulated data for 42 sites over six years, with 10 swabs per visit, and two qPCR runs per swab. In total, we simulated and refit 100 data sets. We determined parameters to be estimable if the 95% credible interval from the parameter estimates contained the parameter value used to simulate the data (i.e., by achieving nominal coverage). Additionally, we assessed bias in parameter estimates by looking at the difference between the estimated and known parameter values divided by the absolute value of the known parameter. We fit all simulated data using 60,000 iterations on three chains with a burn-in of 15,000 iterations, 15,000 iterations of adaptation, and a thinning rate of 8.

## Results

**Fig. 2:**
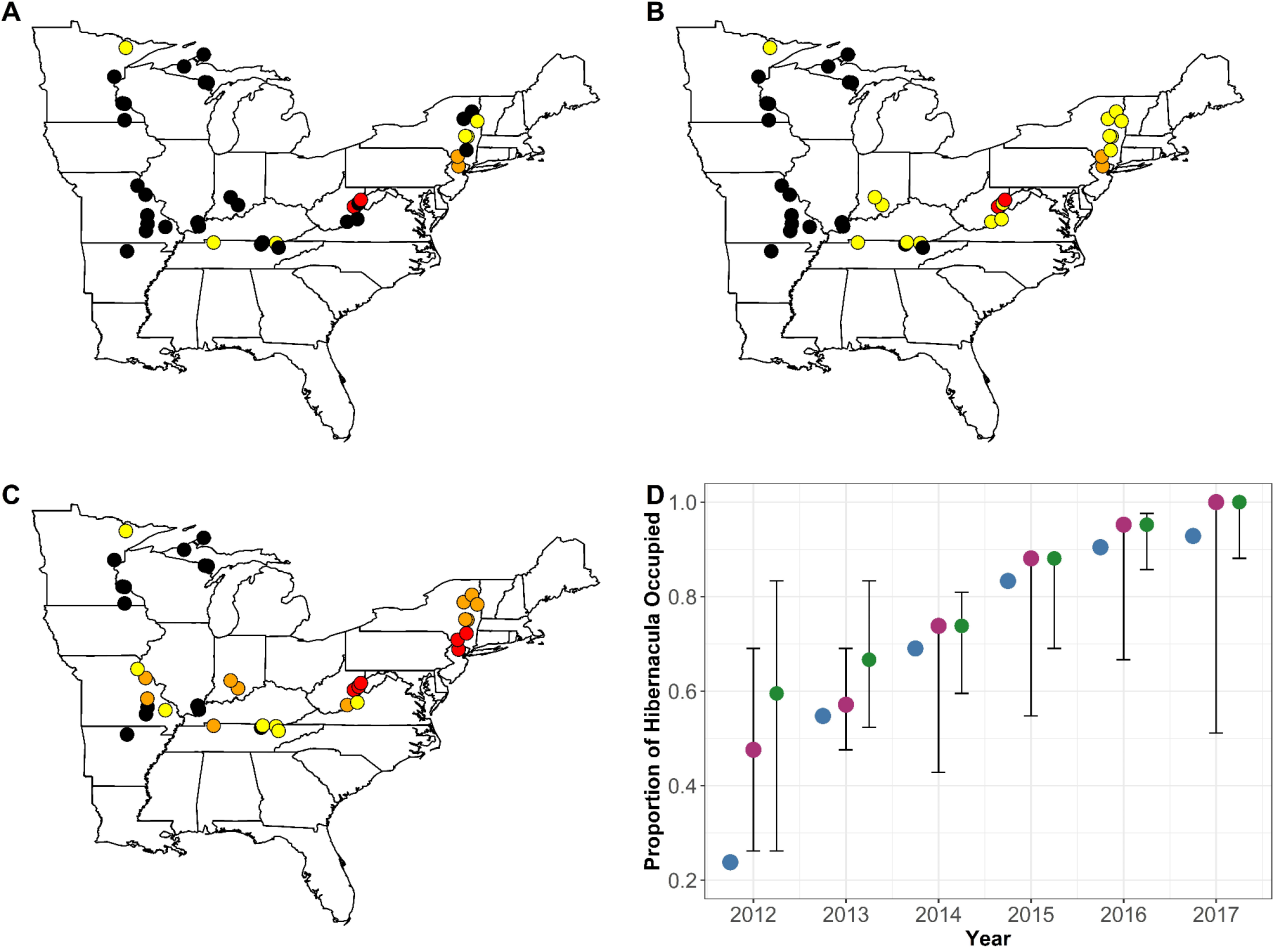
Estimates of *Pseudogymnoascus destructans* presence at the onset of the study (2012) based on naïve occupancy, prevalence, and load (**A**), dynamic occupancy with naïve prevalence and load (**B**), and occupancy, prevalence, and load as predicted by the multi-scale dynamic occupancy hurdle model (**C**). In plots A, B, and C black points represent no pathogen presence and yellow, orange, and red points represent the first, second, and third quantiles of the product of mean prevalence and mean load, respectively. Plot D represents the proportion of hibernacula that have tested positive for or were predicted to be positive for *Pseudogymnoascus destructans* over the study period, with blue points showing naïve occupancy, purple points the dynamic occupancy, and green points the multi-scale dynamic occupancy hurdle model. Error bars around points display the 95% credible interval.

The MS-DOHM model predicted the proportion of *Pd* positive hibernacula to be 3.6% higher than that of the dynamic occupancy model (Range: 0–11.9%) and 11.5% higher than naïve occupancy (Range: 4.8–35.7%) (Fig. 2D). Across all methods, the number of *Pd* positive hibernacula increased every year, with the MS-DOHM and dynamic occupancy model predicting all sampled hibernaculum would be occupied by the end of the study (Fig. 2D). MS-DOHM identified discrepancies in initial year of pathogen arrival at 16.7% sites compared to the dynamic occupancy model and at 52.4% sites compared to the naïve occupancy (Fig. 2A–C). When there was discrepancy between *Pd* arrival, the MS-DOHM always predicted the pathogen to arrive sooner with an average discrepancy of 1.3 years (Range: 1–2) compared to the dynamic occupancy model and 1.4 years (Range: 1–3) compared to naïve occupancy. Discrepancy between naïve and MS-DOHM pathogen arrival excludes three sites that never tested positive, and thus remained negative according to naïve occupancy.

**Fig. 3:**
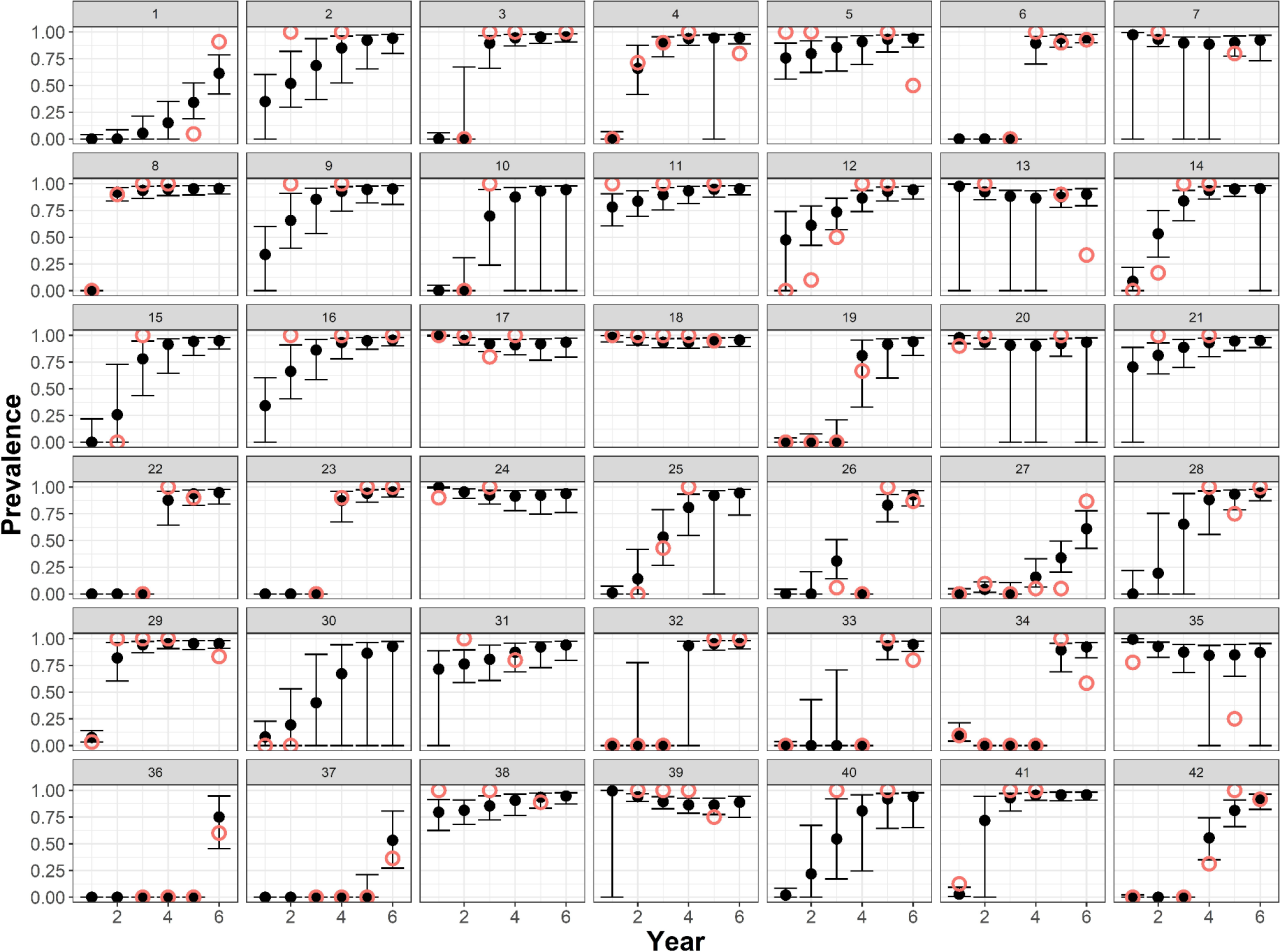
Prevalence of *Pseudogymnoascus destructans* on hibernating *Myotis lucifugus* as predicted by the multi-scale dynamic occupancy hurdle model (solid black points) and 95% credible interval (back lines) across all sites (1– 42) and all years of the study (1– 6 [2012–2017]). Hollow red points indicate naïve prevalence of *Pseudogymnoascus destructans* on hibernating *Myotis lucifugus*. Years without hollow red points indicate years with missing data.

On average, the annual prevalence of *Pd* on hibernating *M. lucifugus* predicted by MS-DOHM did not vary from naïve prevalence (Mean = 0.6%, SD = 16.9%, *p* = 0.67), but there were instances in which model and naïve prevalence differed substantially (Range: -48.1–59.9% [Fig. 3]). Once *Pd* colonized a hibernaculum, the MS-DOHM model predicted near maximum levels of prevalence would be reached in no more than three years. The MS-DOHM never predicted a substantial decline in site-level prevalence for *M. lucifugus* even in hibernacula with near 100% prevalence at the onset of the study. The MS-DOHM predicted greater *Pd* loads on colonies of hibernating *M. lucifugus* than were observed in naïve *Pd* load estimates (Mean = 0.04 ng, SD = 0.04 ng, *p* < 0.001). While *Pd* loads on colonies of *M. lucifugus* were generally found to increase annually, there were instances in which the inverse occurred. All colonies that saw a decrease in mean pathogen load were colonized on or before the first year of the study (2012). There was no correlation between mean colony pathogen load and colony prevalence.

For both the MS-DOHM and the dynamic occupancy model, we found that fixed effects of year (time since first sampling) and distance from *Pd* positive hibernacula in the previous year to be the best predictors of *Pd* colonization of hibernacula, with sites closer to *Pd* positive hibernacula more likely to be colonized in the next time step (Table 1). Differences occurred in model parameter estimates related to pathogen persistence at the hibernaculum level, with MS-DOHM and the dynamic occupancy model predicting increased probability of hibernacula level pathogen persistence into the next winter with greater *Pd* load from the previous year and cave hibernaculum type, respectively (Table 1). The MS-DOHM predicted pathogen prevalence on *M. lucifugus* within hibernacula to be positively associated with the number of susceptible bats (including, *M. septentrionalis*, *P. subflavus*, and *M. sodalis*) and the number of years since *Pd* colonization (Table 1). Initial pathogen loads on *M. lucifugus* colonies was found to be higher in hibernacula which had been colonized by *Pd* for more years, and at the bat level, pathogen load was positively correlated with hibernation day (Table 1). Swab pathogen load was the only covariate used to explain probability of detection, with detection being positively correlated with *Pd* load (Appendix C). Bayesian p-values from the two models, MS-DOHM: 0.66 and dynamic occupancy: 1.0, suggest that although both models had the same data source, only the MS-DOHM fit the data.

**Table 1:** Estimates of the posterior mean, lower 95% credible interval (LCI), and upper 95% credible interval (UCI) for fixed effects of the multi-scale dynamic occupancy hurdle model used to estimate *Pseudogymnoascus destructans*(*Pd*) occupancy, prevalence, and load in North America. Estimates are for the initial occupancy (ψ), colonization (γ), persistence (ε), and load quantity ($\:\mu\:$) of *Pd* at the hibernacula (*h*) and Bat (*b*) scale.

	Mean	LCI	UCI
ψ^h^ Distance to Pathogen	-2.18	-4.01	-0.71
$\:{{\upgamma\:}}^{h}$ Year	1.32	0.33	2.47
$\:{{\upgamma\:}}^{h}$ Distance to Pathogen	-2.45	-4.13	-1.04
$\:{{\upgamma\:}}^{h}$ Distance to Pathogen * Year	1	0.25	1.98
$\:{{\upepsilon\:}}^{h}$ Load	0.56	0.2	1.01
ψ^*b*^ Year of Detection	0.56	0.2	1.01
$\:{{\upgamma\:}}^{b}$ Number of Species	0.94	0.46	1.47
$\:{{\upgamma\:}}^{b}$ Year	1.01	0.33	1.77
$\:\mu\:$^h^ Year of Detection	0.3	-0.02	0.66
$\:\mu\:$^*b*^ Hibernation Days	0.45	0.26	0.64

### Simulation

Across all simulations of the MS-DOHM parameter values used to simulate data were captured by the 95% credible interval of the estimated parameter 94.3% of the time, with individual parameter estimability ranging from 79 to 100%. The median relative bias of estimated parameters was − 1% and ranged from − 0.3 to -1.9% (Appendix B). This level of bias and estimability suggests that the MS-DOHM is capable of accurately and precisely estimating the parameter effects.

## Discussion

Incorporation of information at multiple scales, including all qPCR runs, all sampled *M. lucifugus*, and pathogen load, in the MS-DOM modeling framework improved our understanding of pathogen dynamics and led to earlier pathogen detection compared to the dynamic and naïve occupancy results. The MS-DOM modeling framework provides an alternative to current methods that designate low-level qPCR results as “inconclusive,” which can cause confusion among practitioners and lead to delayed decisions in WNS response and management. By incorporating Ct scores in the MS-DOM modeling framework, we are able to better inform pathogen presence (i.e., occupancy) at the site level that provides practitioners with a more accurate indication of *Pd* invasion at a site. Early pathogen detection can be critical for rapid response to mitigate disease impacts, slowing the pathogen spread, and helping guide surveillance efforts and conservation response^2,51,52^.

The MS-DOHM predicted patterns of pathogen prevalence in hibernating *M. lucifugus* colonies to follow a pattern consistent with previous studies, seeing a peak in prevalence within two to three years of pathogen arrival and sustaining this peak over time^19,32,53^. Our results indicated that naïve estimates of mean *Pd* load on winter colonies at hibernacula were significantly less than the mean *Pd* loads predicted by the MS-DOHM. Underestimation of mean *Pd* loads on bats at hibernacula could lead to poor estimates of site-level disease severity or stage of pathogen invasion. Like other studies, we found mean *Pd* loads on winter colonies increased during winter and for several years after pathogen arrival, with some instances of mean pathogen loads decreasing in hibernacula that had been colonized on or before the onset of the study^14,19,54,55^. Decreases in mean *Pd* load could suggest that bats in some *M. lucifugus* colonies are developing resistance to *Pd*^55,56^.

Like prior studies, we found the probability of pathogen occupancy at hibernacula increased with proximity to *Pd* positive hibernacula and time^44,45^. The MS-DOHM model indicated that pathogen prevalence was positively correlated with year of first pathogen detection at a site, which is consistent with other studies that observed an increase in *Pd* prevalence with time since pathogen invasion^19,32,53^. The effect of species composition on site-level *Pd* presence and disease mortality have been investigated but a significant correlation has not been demonstrated^45,57^. We found that the presence of other WNS-susceptible species was positively associated with *Pd* prevalence in *M. lucifugus*, which is consistent with findings that interspecies contact is not uncommon in hibernacula^46^. The MS-DOHM indicated that days since the onset of hibernation was positively associated with pathogen load on bats which is consistent with previous studies that show pathogen loads increase as the infection progresses over winter^19^.

We present a modeling framework, MS-DOHM, that provides an improved and more robust method for designating *Pd* invasion by integrating nested scales of *Pd* detection (swab, bat, site), which resulted in earlier estimates of pathogen arrival and improved knowledge of pathogen prevalence and load in years when sampling did not occur. As *Pd* continues to spread west and north through the North American continent, the ability to detect *Pd* at low levels to identify pathogen invasion and spread continues to be a challenge. However, utilization of this model could better identify bat colonies at-risk of imminent *Pd* invasion and allow for a better understanding of disease progression and earlier disease treatment intervention, such as vaccine and topical probiotics^58,59^. Given the widespread issue of interpreting low-level qPCR positives as “inconclusive” in many disease systems, adoption of this modeling framework could help improve interpretation of otherwise confusing results, and help facilitate rapid response.

## Electronic supplementary material

Below is the link to the electronic supplementary material.

Supplementary Material 1

Supplementary Material 2

Supplementary Material 3

Supplementary Material 4

## Data Availability

The datasets and code used for this study are provided under supplementary information.
