# Supplementary material for: A novel method for estimating pathogen presence, prevalence, load, and dynamics at multiple scales
Source: Sci Rep. 2025 Mar 19;15:9423. doi: 10.1038/s41598-025-93865-x (PMC11923299; doi:10.1038/s41598-025-93865-x)
Supplement: Supplementary file 1 — Supplementary Material 1 [file 41598_2025_93865_MOESM1_ESM.docx]

**A novel method for estimating pathogen presence, prevalence, load, and dynamics at multiple scales**

John F. Grider^1,2^, Bradley J Udell^3^, Brian E. Reichert^3^, Jeffrey T. Foster^4^, William L. Kendall^5^, Tina L. Cheng^6^, Winifred F. Frick^6,7^

1. Colorado Cooperative Fish and Wildlife Research Unit, Colorado State University, 1484 Campus Delivery, Fort Collins, Colorado 80523 U.S.A

2. Colorado Parks and Wildlife, Wildlife Health Program, 4330 Laporte Avenue, Fort Collins, Colorado 80521, USA

3. U.S. Geological Survey, Fort Collins Science Center, 2150 Centre Avenue, Fort Collins, CO 80526, U.S.A

4. Pathogen and Microbiome Institute, Northern Arizona University, Flagstaff, AZ U.S.A

5. U. S. Geological Survey, Colorado Cooperative Fish and Wildlife Research Unit, Colorado State University, 1484 Campus Delivery, Fort Collins, Colorado 80523 U.S.A

6. Bat Conservation International, 500 North Capital of Texas Highway, Building 1, Austin, TX 78746, U.S.A

7. Department of Ecology and Evolution, University of California, 130 McAllister Way, Santa Cruz, Santa Cruz, CA 95060, U.S.A.

Corresponding author

John F. Grider

jack.grider@state.co.us


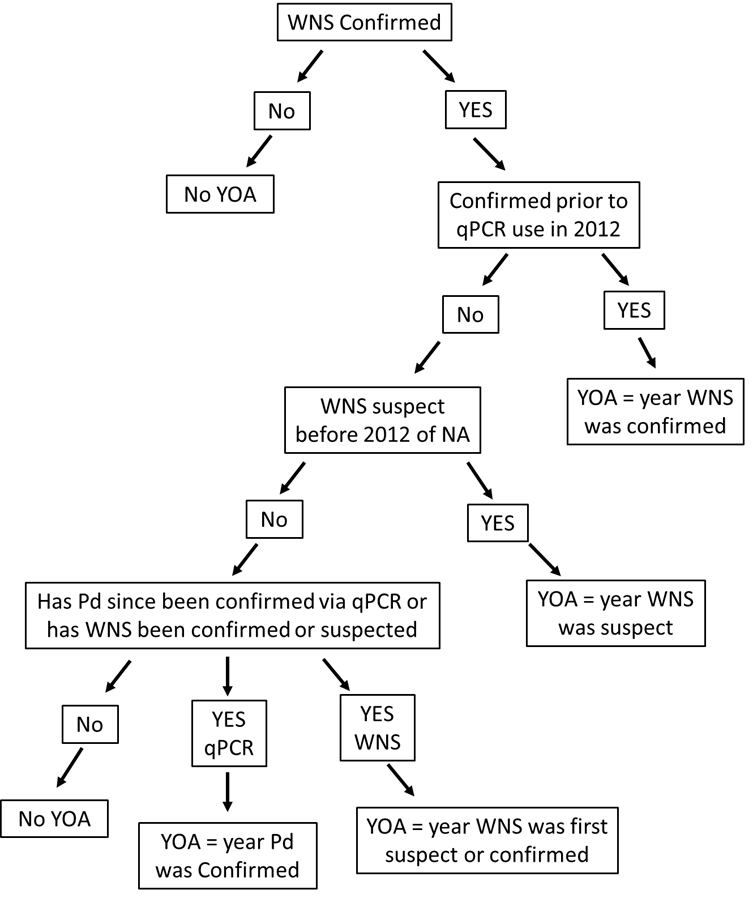


**Appendix A**: Decision tree used to estimate year of *Pseudogymnoascus destructans* (Pd) year of arrival (YOA). The decision tree assigns YOA based on information from Pd detected by quantitative real-time PCR (qPCR) and white-nose syndrome (WNS) confirmed or suspected based on the U.S. Geological Survey (USGS) spread map. In many cases, WNS “suspect” is equivalent to a qPCR positive but could also be signs of WNS. WNS confirmed indicates a case where lesions were observes via histological analysis. The tree begins with only cases in which WNS was confirmed or suspected because prior to 2012 qPCR Pd surveillance was not available.


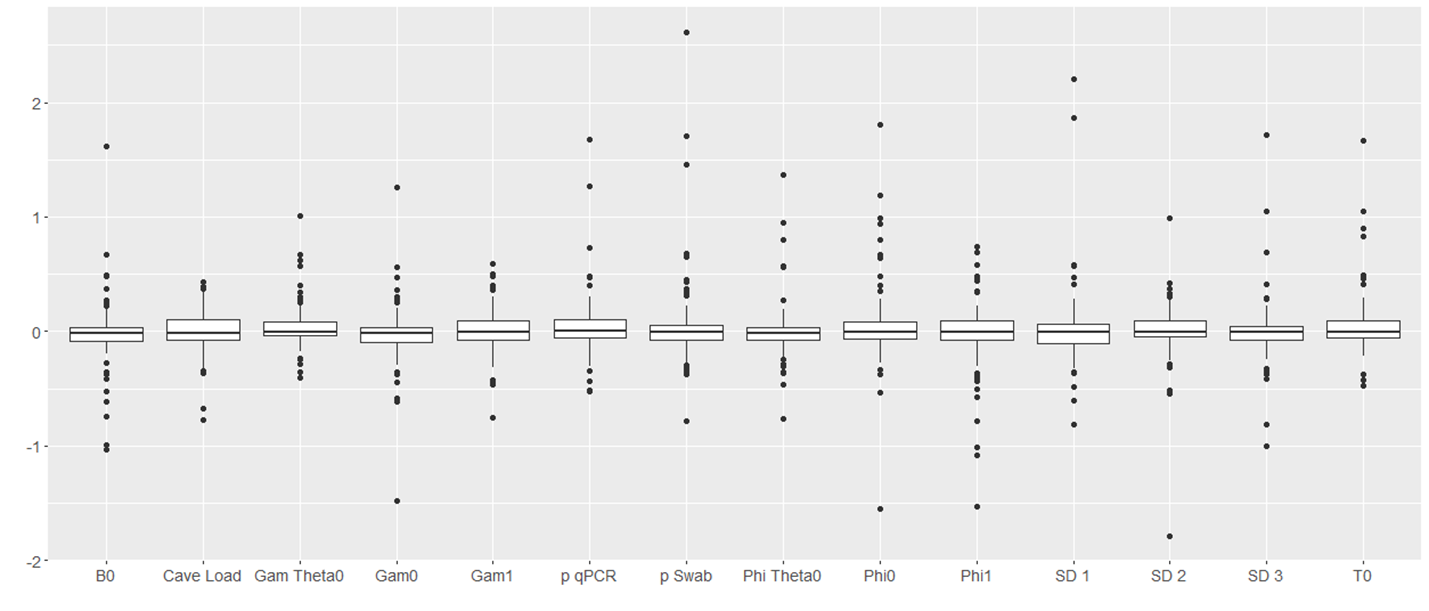


**Appendix B**: Bias of every parameter estimate based off simulating 100 datasets with known parameters and refitting simulated data to the multi-scale dynamic occupancy hurdle model (MS-DOHM). The bias was determined by taking the difference between the estimated parameter and known parameter divided by the absolute value of the known parameter. A value of zero indicates no bias.


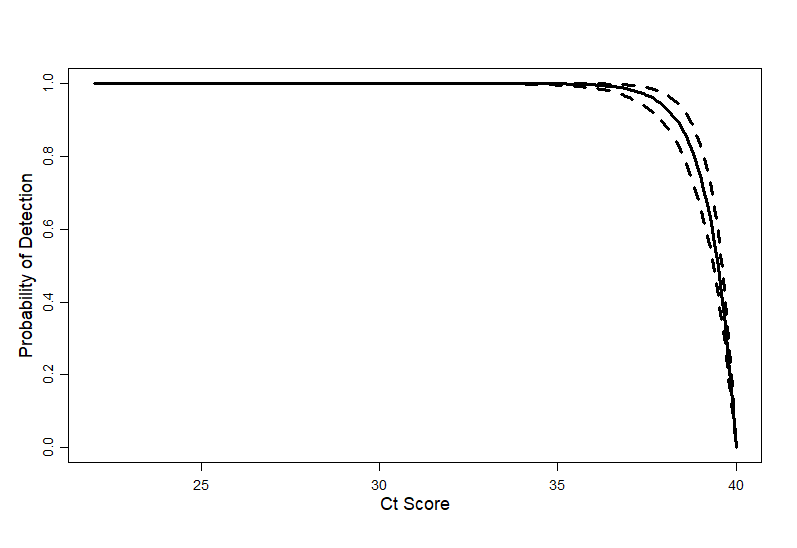


**Appendix C**: Mean (solid black line) and 95% confidence interval (black dashed line) for the probability of detecting the fungal pathogen *Pseudogymnoascus destructans on* hibernating little brown bats (*Myotis lucifugus*) in relation to the quantitative real-time PCR cycle threshold (Ct).
